# Supplementary material for: Functional Optimization in Distinct Tissues and Conditions Constrains the Rate of Protein Evolution
Source: Mol Biol Evol. 2024 Oct 21;41(10):msae200. doi: 10.1093/molbev/msae200 (PMC11523136; doi:10.1093/molbev/msae200)
Supplement: msae200_Supplementary_Data [file msae200_supplementary_data.zip › SupplementaryTables_Legends.pdf]

Table S1
The joint influence of gene expression across tissues on protein evolutionary rates in animals.

Table S2
The relationship of tissue- or cell-type-specific expression with protein evolutionary rates for all datasets and with protein functional optimality for *H. sapiens*, *A. thaliana*, and *E. coli*. The average expression levels of synaptic genes across cell-types of *M. musculus* and *D. melanogaster* brains and of growth marker genes across samples from *Z. mays*, *A. thaliana*, *G. max*.

Table S3
Expression – evolutionary rate correlations for groups of human genes associated with specific Gene Ontology categories.

Table S4
The Gene Ontology terms upregulated in the cell-types and tissues with stronger expression-evolutionary rate correlations, obtained by gene set enrichment analyses.

Table S5
Information about gene expression datasets used in this work.

Table S6
The data on evolutionary rates across genes of *H. sapiens*, *M. musculus*, *D. melanogaster*, *C. elegans*, *Z. mays*, *A. thaliana* and *G. max*.

Table S7
Lists of marker genes for different growth stages in plants and synaptic genes in animals.

Table S8
The complete dataset of catalytic rates of the enzymes used in this work.

Table S9
The data on catalytic rates, evolutionary rates, and expression values for enzymes from *H. sapiens*, *A. thaliana*, and *E. coli*.

Table S10
The relationship between the catalytic rates of the enzymes and the rate of their evolution.
