# Supplementary material for: Functional Optimization in Distinct Tissues and Conditions Constrains the Rate of Protein Evolution
Source: Mol Biol Evol. 2024 Oct 21;41(10):msae200. doi: 10.1093/molbev/msae200 (PMC11523136; doi:10.1093/molbev/msae200)
Supplement: msae200_Supplementary_Data [file msae200_supplementary_data.zip › TableS5_ExpressionDatasets.pdf]

Organism	Description of samples	Ref.	Sequencing technique	Comments
Homo sapiens	53 tissues	(Mele, et al. 2015)	RNA-seq	Tissue-specific gene expression data was obtained from GTEx portal at www.gtexportal.org (release v7). Specifically, we used the median of TPM-normalized expression values across samples from different donors.
Mus musculus	13 tissues	(Söllner, et al. 2017)	RNA-seq	Tissue-specific RPKM-normalized expression values were obtained from https://www.ebi.ac.uk/biostudies/arrayexpress/studies/E-MTAB-6081. Expression values were averaged across several samples available for each tissue.
Drosophila melanogaster	10 tissues of the larvae stage	(Leader, et al. 2018)	RNA-seq	Tissue-specific FPKM-normalized expression values for *D. melanogaster* larvae were obtained from FlyAtlas 2 (https://motif.mvls.gla.ac.uk/FlyAtlas2) via the URL request interface.
Caenorhabditis elegans	7 tissues of the larvae stage L2	(Spencer, et al. 2011)	microarray	Tissue-specific expression values for *C. elegans* larvae (stage L2) were downloaded from https://www.vanderbilt.edu/wormdoc/wormmap/Welcome.html. The original transcriptome was quantile normalized, log2 transformed, and averaged over three replicas for each tissue. For consistency with other datasets used in this work, we transformed *C. elegans* expression values from the log2 to linear scale.
Mus musculus	207 cell-type clusters of the mouse nervous system cells	(Zeisel, et al. 2018)	scRNA-seq	Expression profiles and metadata for individual cells were downloaded from http://mousebrain.org/adolescent/downloads.html. Majority of scRNA-seq samples was sequenced with the 10X Genomics Chromium Single Cell Kit Version 1, while ~5% with the Kit Version 2. To avoid any potential biases and batch effects, related to the different procedures and the usage of different kits, we removed all data obtained with the V2 kit. We combined cells sequenced with the V1 kit into cell-type clusters according to the original annotation.
Mus musculus	404 cell-type clusters of the mouse brain cells	(Saunders, et al. 2018)	scRNA-seq	Expression profiles and metadata for cell-type clusters were obtained from http://dropviz.org/.
Mus musculus	203 cell-type populations defined by anatomical and genetic identities	(Sugino, et al. 2019)	RNA-seq	TPM-normalized expression values for cell-type populations and their annotation were downloaded from https://neuroseq.janelia.org/. In cases where several replicates were available for a given cell-type, we averaged the expression levels across the replicates.

Drosophila melanogaster	172 cell-type clusters of the adult fly brain cells collected at different developmental stages	(Davie, et al. 2018)	scRNA-seq	Expression profiles and metadata for individual cells were downloaded from https://www.ncbi.nlm.nih.gov/geo/query/acc.cgi?acc=GSE107451. Davie *et al*. sequenced cells from the adult fly brains collected at 8 ages ranging from 0 to 50 days old and grouped them into 87 clusters based on marker genes. To avoid the age-dependency of cell transcriptomes within each cluster, we split each original cell-type cluster by fly age. We also removed cells collected at day 0 to minimize the effects associated with the transition from the pupae to the adult stage.
Mus musculus	Region-specific expression for 674 regions of the mouse brain	(Lein, et al. 2007)	*in situ* hybridization	Region-specific expression data was obtained from the Allan Brain Institute Atlas at http://help.brain-map.org/display/mousebrain/api. For the analysis we used the "expression energy", i.e., the ISH intensity across voxels of the corresponding brain area. To remove regions with the low-quality expression profiles we kept in the analysis only brain structures with more than 17000 detected genes.
Mus musculus	6 mouse tissues at 14 different developmental time points	(Cardoso-Moreira, et al. 2019)	RNA-seq	Tissue and age-specific TPM-normalized expression values were downloaded from https://www.ebi.ac.uk/gxa/experiments/E-MTAB-6798/Downloads?ref=aebrowse. We did not use expression data for the Cerebellum/Hindbrain tissue as it was not measured at early embryonic time points. For other samples we averaged expression levels if several replicas were available for a tissue at a specific developmental stage.
Arabidopsis thaliana	79 tissues	(Klepikova, et al. 2016)	RNA-seq	Tissue-specific DESeq2-normalzied expression values were obtained from the authors. We averaged expression levels over two replicates available per tissue.
Zea mays (Corn)	92 tissues	(Stelpflug, et al. 2016)	RNA-seq	Tissue-specific TPM-normalized expression values were obtained from https://www.ebi.ac.uk/gxa/experiments/E-MTAB-4342/Downloads.
Glycine max (soybean)	25 tissues	(Shen, et al. 2014)	RNA-seq	Tissue-specific TPM-normalized expression values were obtained from https://www.ebi.ac.uk/gxa/experiments/E-MTAB-4270/Downloads.
E. coli	*E coli* at the log phase growth	(McClure, et al. 2013)	RNA-seq	Expression data for *E. coli* at the log-phase growth stage was downloaded from https://www.ncbi.nlm.nih.gov/geo/query/acc.cgi?acc=GSE45443. We averaged expression values across all growth conditions in the nutritionally rich media.
